# Study on Static Mechanical Properties and Numerical Simulation of Coral Aggregate Seawater Shotcrete with Reasonable Mix Proportion

**DOI:** 10.3390/ma17102353

**Published:** 2024-05-15

**Authors:** Yuxuan Peng, Liyuan Yu, Wei Li, Tao Zhang, Linjian Ma, Dongyang Wu, Changan Wu, Linjie Zhou

**Affiliations:** 1 State Key Laboratory of Intelligent Construction and Healthy Operation and Maintenance of Deep Underground Engineering, China University of Mining and Technology, Xuzhou 221116, China; yxpeng134885@163.com (Y.P.); tbh259@cumt.edu.cn (W.L.); ts17030057a3tm@cumt.edu.cn (T.Z.); 15651352723@163.com (D.W.); ts22030236p31@cumt.edu.cn (C.W.); 18168215683@163.com (L.Z.); 2 State Key Laboratory of Explosion Shock Prevention and Mitigation, Army Engineering University of PLA, Nanjing 210007, China; patton.4400@163.com; 3 Yunlong Lake Laboratory of Deep Underground Science and Engineering, Xuzhou 221008, China

**Keywords:** coral aggregate seawater shotcrete, particle flow code, mix proportion, static mechanical properties, orthogonal experiment

## Abstract

This study aims to explore the static mechanical characteristics of coral aggregate seawater shotcrete (CASS) using an appropriate mix proportion. The orthogonal experiments consisting of four-factor and three-level were conducted to explore an optimal mix proportion of CASS. On a macro-scale, quasi-static compression and splitting tests of CASS with optimal mix proportion at various curing ages employed a combination of acoustic emission (AE) and digital image correlation (DIC) techniques were carried out using an electro-hydraulic servo-controlled test machine. A comparative analysis of static mechanical properties at different curing ages was conducted between the CASS and ordinary aggregate seawater shotcrete (OASS). On a micro-scale, the numerical specimens based on particle flow code (PFC) were subjected to multi-level microcracks division for quantitive analysis of the failure mechanism of specimens. The results show that the optimal mix proportion of CASS consists of 700 kg/m^3^ of cementitious materials content, a water–binder ratio of 0.45, a sand ratio of 60%, and a dosage of 8% for the accelerator amount. The tensile failure is the primary failure mechanism under uniaxial compression and Brazilian splitting, and the specimens will be closer to the brittle material with increased curing age. The Brazilian splitting failure caused by the arc-shaped main crack initiates from the loading points and propagates along the loading line to the center. Compared with OASS, the CASS has an approximately equal early and low later strength mainly because of the minerals’ filling or unfilling effect on coral pores. The rate of increase in CASS is swifter during the initial strength phase and decelerates during the subsequent stages of strength development. The failure in CASS is experienced primarily within the cement mortar and bonding surface between the cement mortar and aggregate.

## 1. Introduction

Owing to the ongoing advancement of islands and reefs in proximity to the mainland, incorporating coral debris and coral sand into construction materials for infrastructure such as roads, docks, slope protection, and buildings has emerged as the predominant selection for island reef projects. This preference is attributed to its cost-effectiveness, durability, and workability characteristics [1,2,3,4,5,6,7]. Specifically, coral serves as a natural lightweight aggregate, displaying a comparatively elevated water-absorption rate and possessing traits for both water absorption and release when contrasted with standard coarse aggregates made from crushed stone [8]. The surface roughness of coral aggregates surpasses that of traditional lightweight aggregates, resulting in an increased frictional interaction between the aggregate and cement paste. This, in turn, strengthens the interfacial bond between the cement paste and matrix [9,10]. Moreover, salt compounds like chloride ions and sulfate ions present in coral contribute to the initial strength advancement of concrete [11,12]. However, shotcrete serves as an essential means and method for structural support during the initial phases of construction [13], and it needs such superior early strength. Exploring the static mechanical characteristics of CASS with a reasonable mix proportion is essential to alleviate pressure on the supply of raw materials for the initial support undertaking of island reef shotcrete projects.

It is worth noting that there are no universally applicable mix proportion design criteria specifically tailored for coral aggregate concrete (CAC). Earlier studies have indicated that various factors, such as the cementitious materials content, the water–binder ratio, and the sand ratio, influence the workability and mechanical characteristics of CAC [14,15]. Moreover, the accelerator amount is a crucial parameter that necessitates consideration when applying CAC as shotcrete [16,17]. Nevertheless, employing the conventional method of single-factor control to ascertain the impacts of all parameters on the optimal mix proportion design can be both time-consuming and challenging. Orthogonal experiment is a commonly used method for multi-factor experiments and provides an excellent solution to the challenges linked with the traditional single-factor control approach [18,19,20]. Based on the orthogonality, only a handful of representative experiments are necessary to find the optimal mix proportion.

On the one hand, machinery and laborers must be capable of sustaining drive progression before the shotcrete attains maximum strength. However, the existing experimental data concerning the time-dependent material properties of shotcrete is somewhat limited [21,22], with many of the data sets being outdated. On the other hand, coral aggregates exhibit variations in morphology and porosity when compared to typical natural and lightweight aggregates. This disparity results in a significant structural and performance distinction between CASS and OASS [23]. Hence, there is a need to examine the static mechanical characteristics of CASS at various curing ages and conduct a comparative analysis with OASS to verify the validity of CASS mix proportion [24].

Meanwhile, to address the limitations of laboratory tests that fail to capture the complete deformation and damage process of structures in real time, the remarkable advancements in computer technology have facilitated the growing adoption of numerical simulations for investigating the static mechanical properties of concrete [25]. Concrete is a composite material with heterogeneous phases, including both coarse and fine aggregates, water, cement, and additional constituent elements [26]. It is noteworthy to understand that the meso-modeling approach posits concrete as a composite material with three phases: aggregate, interfacial transition zone, and cement mortar components. This methodology has gained substantial application and has been extensively developed in various research studies [27,28,29,30]. Meso-modeling offers valuable insights into the macroscopic behavior and performance of materials. In contrast to the meso-modeling approach, the discrete element method (DEM) holds distinct advantages for more accurately reproducing grain shapes [31,32,33] and their interactions [34,35,36,37]. The software particle flow code (PFC 6.0) based on the discrete element method has gained significant popularity within geo-materials due to its properties similar to the fundamental assumptions and construction principles of geo-materials [38,39,40]. Among them, Haeri and Sarfarazi utilized the particle flow code in two dimensions (PFC2D) to investigate the damage mechanism of concrete under tensile failure and suggested that a modified tension test can be a proper test for the determination of the tensile strength of concrete in absence of direct test [41]; Lian et al. presented a numerical model utilizing the PFC2D to estimate the mechanical characteristics of porous concrete [42]; Song et al. elucidated the mechanical response of concrete subjected to multi-level cyclic loading and unloading based on particle flow code in three dimensions (PFC3D) [38]. Nevertheless, most prior investigations had focused on the macro-scale failure mode of the specimen without delving into the micro-scale failure mechanisms at a finer scale.

Various models have been employed to simulate aggregates in concrete materials with the growing adoption of numerical simulations. These include 2D circular models [43], 2D random polygon models, 3D spherical models [44], 3D ellipsoid models [45], 3D random convex polyhedron models [46,47], as well as non-convex polyhedron models [48]. However, these studies have simplified the coarse aggregate by representing it as clusters of particles resulting from overlapping a limited number of individual particles. Unfortunately, this approach fails to reproduce the influence of the rough porousness characteristic of coral aggregate on the mechanical properties of specimens. Moreover, the densification effect resulting from filling coral aggregate pores with small mineral particles has yet to be involved.

This study conducted four-factor and three-level orthogonal experiments to explore the optimal mix proportion of CASS. First, the AE technique was used to analyze the compressive failure mechanism and damage phenomenon of CASS with optimal mix proportion. The digital image correlation (DIC) technique was used to analyze the splitting failure mechanism and deformation characteristics of CASS with optimal mix proportion. The static mechanical properties of CASS and OASS at different curing ages were compared to verify the validity of the CASS mix proportion. The minerals’ filling or unfilling effect on coral pores was systematically analyzed. After that, a novel three-dimensional numerical model based on PFC was proposed to investigate the static mechanical properties and failure mechanism of CASS. Multi-level division and quantitative analysis of microcracks generated between various mineral particles calibrated with different mechanical parameters were carried out. Finally, the failure mechanism of CASS was quantitatively analyzed with the results of numerical simulation and experimental microstructure observation.

## 2. Materials and Experiment Procedure

### 2.1. Raw Materials and Mix Proportion

**Figure 1:**
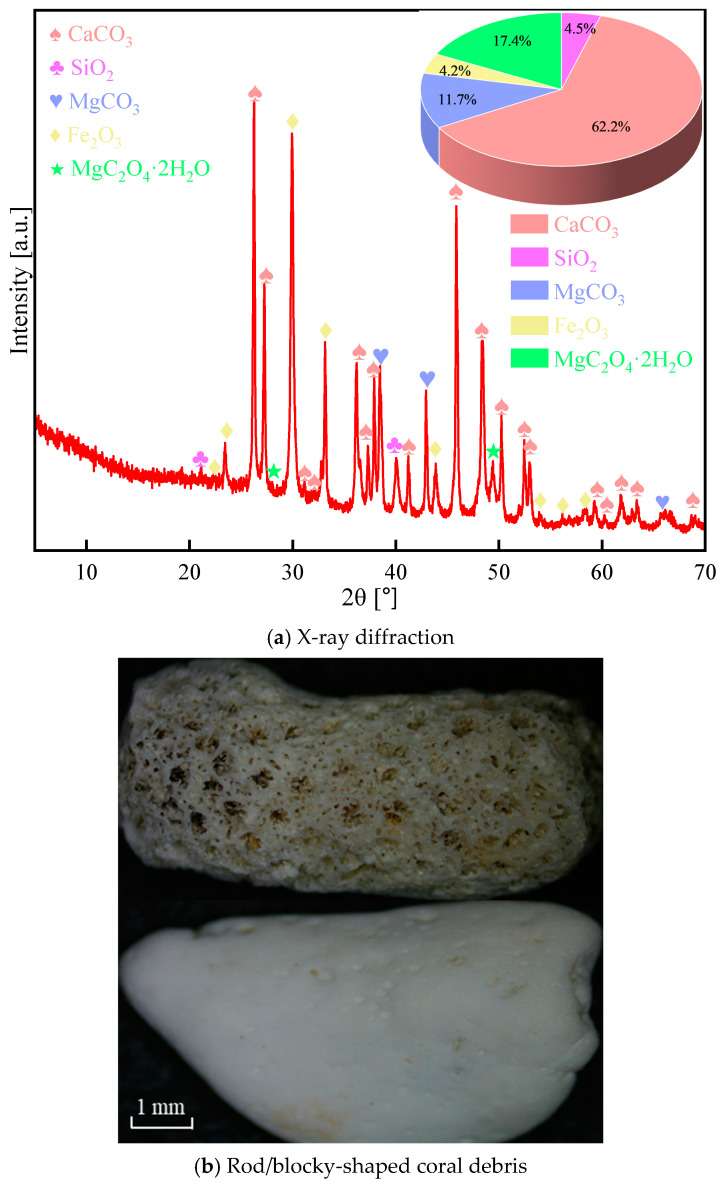
Microstructure and mineral composition of coral in its natural state.

**Figure 2:**
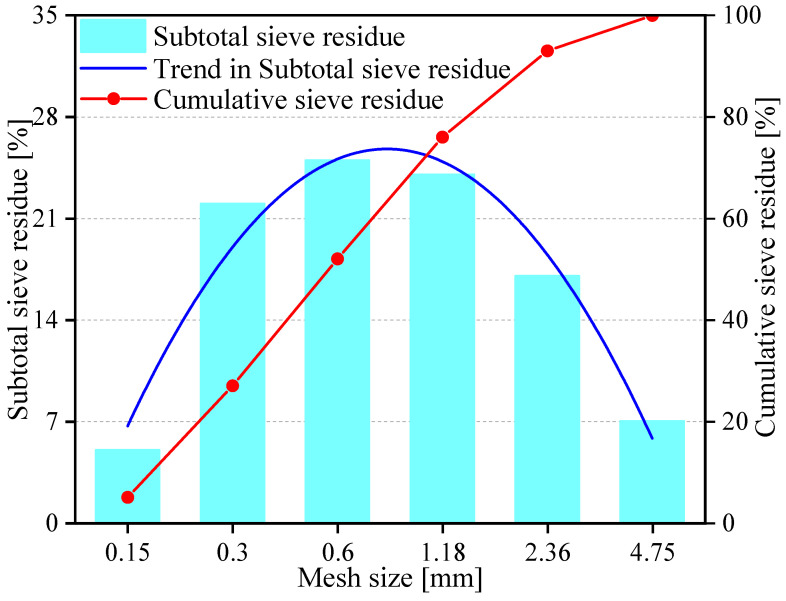
Coral sand particle grading curve.

Both coral debris and coral sand, serving as coarse and fine aggregates, were directly obtained from the South China Sea islands. Different from natural quartz sand or mineral rocks originating from physical or chemical processes, coral debris and coral sand predominantly constitute the remains of hermatypic polypary corals and shells. These materials mainly arise as biologically derived sediments. Coral debris and coral sand share an identical chemical composition, consisting predominantly of 62.2% calcium carbonate, along with iron, magnesium, and silicon ions (Figure 1a). Microscopic examination of the coral surface, as depicted in Figure 1b, reveals a visual rough and porous texture. This qualitative assessment is indicative of the coral’s natural structure, which differs significantly from that of conventional natural aggregates such as those found in river beds, which results in its diminished physical and mechanical properties. However, coral sand has a relatively smooth surface due to its smaller particle size and significant weathering. In order to ensure optimal fluidity and sprayability of CASS, the particle size of coral debris is controlled within the range of 5–10 mm. Figure 2 depicts the distribution of grain sizes in coral sand, exhibiting a fineness modulus of 2.88. The fundamental physical characteristics of both coarse and fine aggregates, such as bulk and apparent densities, porosity, water absorption, cylindrical compressive strength, and saturated surface dry moisture content, have been examined in accordance with the Chinese standards GB/T 14684-2011 [49]. The corresponding test outcomes are available in both Table 1 and Table 2.

**Figure 3:**
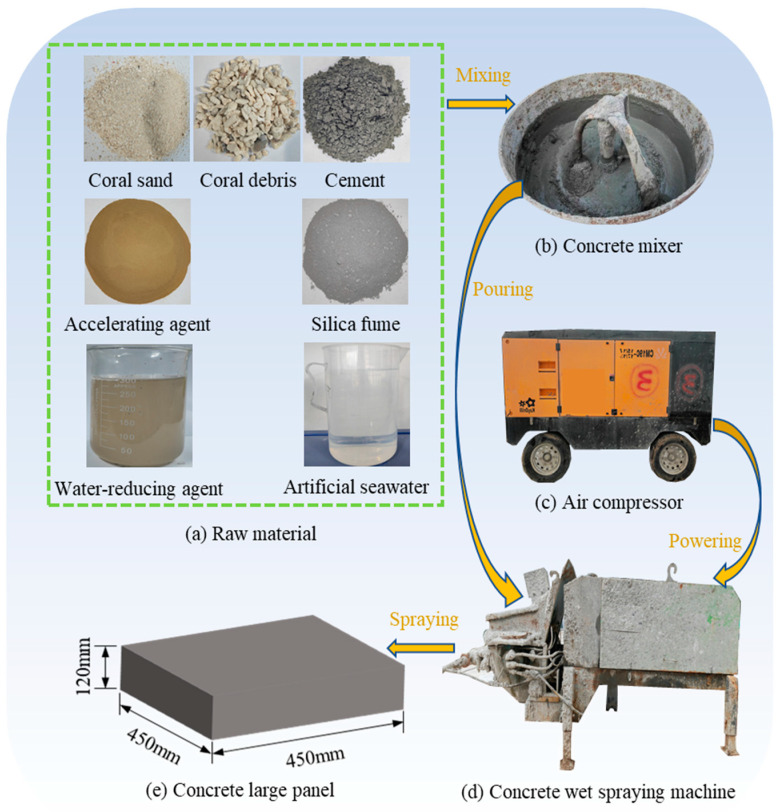
Schematic of the large-panel specimen-making process.

As shown in Figure 3a, all the raw materials for the preparation of CASS are presented. Among these components, the cement used is 42.5 ordinary Portland cement (P.O.42.5) from Xuzhou Zhonglian Cement Co., Ltd. (Xuzhou, China). Silica fume and water-reducing agents consist of ultra-fine wollastonite powder and a high-performance water-reducing agent with weak alkalinity, provided by Shanghai Chenqi Chemical Technology Co., Ltd. (Shanghai, China). The alkali-free liquid accelerator is sourced from Laiyang Keyu Construction Admixture Factory (Laiyang, China). Additionally, artificial seawater was prepared following the American ASTM D1141-2003 standard [50], and its chemical composition is detailed in Table 3. In this study, all chemicals used for artificial seawater preparation were obtained from Nanjing Reagent Factory with a purity of analytical grade.

In this study, the mixed proportions of the coral aggregate seawater shotcrete (CASS) were designed following the guidelines of the national standards JGJ51-2002 [51] and JGJ/T 372-2016 [52]. These standards are equivalent to and have been aligned with the principles of the European Standard (EN) 12620:2012 [53] for aggregates for concrete and the American Concrete Institute (ACI) Guide for Selecting Proportions for Normal, Heavyweight, and Mass Concrete (ACI 211.1R-19), respectively. This alignment ensures that our findings are applicable within the national context and relevant to the broader international community. However, owing to the notable distinction between coral aggregate and conventional aggregate, a set of compressive strength tests focusing on four factors, cementitious material content, water–binder ratio, sand ratio, and accelerator quantity, was conducted. Through extensive mix proportion trials, it was revealed that specimens cured for 28 days exhibited relatively higher strength with a cementitious material content of 700 kg/m^3^, a water–binder ratio of 0.45, a sand ratio of 50%, and an accelerator amount of 6%. Building upon this information, an orthogonal experiment employing a four-factor and three-level design (refer to Table 4) was executed to ascertain the optimal mix ratio for CASS. The experiment comprised nine mix proportions, detailed in Table 5.

### 2.2. Specimen Preparation and Basic Physical Properties

The sequence of cement, cementitious material, coral sand, coral debris, water reducer, and seawater was employed while stirring raw materials using a concrete mixer with a rotation speed of 300 revolutions per minute. To achieve consistent mixing, half of the seawater was initially added to the mixture and stirred for 60 s, followed by the addition of the remaining half and subsequent mixing for 120 s. As shown in Figure 3, the wet spraying method was used in this study. The wet-mixed concrete was then prepared for spraying by adjusting the air compressor’s pressure to 0.5 MPa, which was used to propel the mixture to the nozzle of the concrete wet-spraying machine and mixed with the accelerator. Lastly, the specimen preparation was completed by spraying the concrete into a 450 mm × 450 mm × 120 mm size mold with an angle of 75° to the ground by opening the nozzle valve. In addition, as shown in Table 6, the slump and rebound rate tests were carried out during specimen preparation. The CASS specimens were demoulded after one day and placed in a curing room with a constant temperature of 20 °C and a relative humidity of 95% for 28 days. The large-panel specimens made by spraying needed to be cored, cut, and polished to gain standard specimens for uniaxial compression tests (UCS) and Brazilian splitting tests (ST).

### 2.3. Quasi-Static Tests

A series of orthogonal experiments, including the uniaxial compression and Brazilian splitting with a loading rate of 0.3 mm/min and 0.05 mm/min, respectively, were conducted on CASS specimens with different mix proportions and curing ages using the DNS 100 electronic universal testing machine at the China University of Mining and Technology. Simultaneously, vaseline was administered at both ends of the specimens to alleviate the impacts of end friction and stress concentration before the application of the uniaxial compression load. Each specimen’s peak compressive strength and tensile strength were recorded and analyzed during the tests to obtain the optimal mix proportion of CASS. In order to verify the validity of the optimal mix proportion and quantify the effect of aggregate type on the macro-mechanical parameters of concrete at various curing ages, the optimal mix proportion of CASS was selected, and the ordinary aggregate seawater shotcrete (OASS) specimens were made with ordinary aggregate instead of coral aggregate.

During the Brazilian splitting tests, the tensile strength *σ*_t_ can be calculated as follows [54]:

(1) $$\sigma_{t}=\frac{2P_{\max}}{\pi DB}$$

where *P*_max_ denotes the applied load maximum, D denotes the diameter of the test specimen, and *B* denotes the thickness of the test specimen.

**Figure 4:**
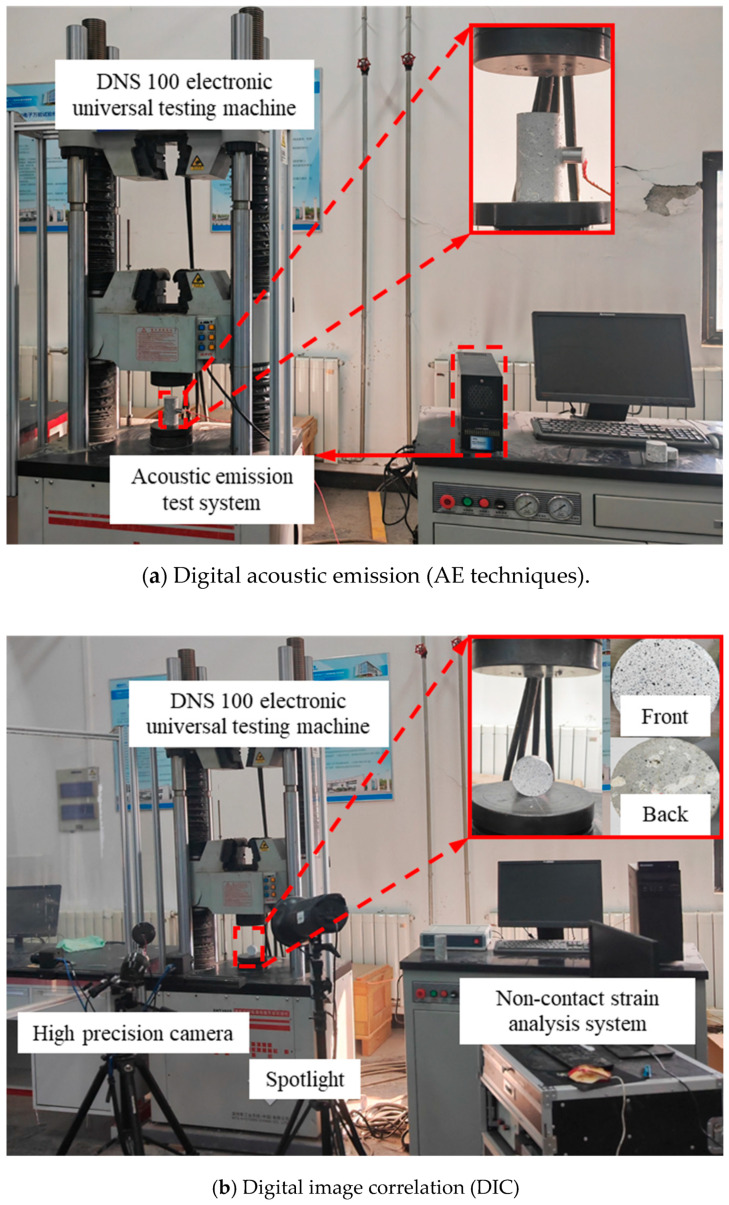
Monitoring system.

In order to clarify the fracture failure mechanism and damage phenomenon of concrete with an optimal mix proportion, a Micro-II AE technique monitoring system developed by American Physical Acoustics Company (PAC) was used to record the changes in AE characteristic signals induced by microcrack development under uniaxial compression, such as counting, energy, and frequency. To precisely capture the AE signals produced by the upper and lower ends of the specimens, the receiving sensor was affixed at the midpoint of each specimen. In the AE monitoring system shown in Figure 4a, the elastic wave emitted by the signal source propagates to the surface of the specimens, causing the surface displacement of the AE sensor and converting the mechanical vibration into electrical signals. It is essential to highlight that, for efficient filtering of environmental noise and enhanced monitoring of cracking information quality, the threshold value was established at 45 dB. To account for the impact of mechanical noise and instrument sensitivity, the acquisition frequency for AE signals was chosen to be 3 MHz. In the Brazilian splitting tests, the duration from crack initiation to propagation and penetration until the specimen failure is brief. Particularly, the period from crack penetration to specimen failure is nearly instantaneous. Therefore, the AE characteristic signal induced by microcrack development in Brazilian splitting tests cannot explain the failure mechanism obviously. However, DIC is a non-contact visual processing method for measuring surface deformation of materials widely used in research related to crack propagation [55,56]. Specifically, DIC enables the observation of non-uniform changes in the macroscopic strain field due to the cumulative expansion of microcracks. Its basic principle involves using an initial reference image as a baseline. The target research area is divided into a network, and the motion of segmented subregions is treated as rigid transformations. Through a predefined matching algorithm, specific subsets of position changes before and after surface deformation are identified. This process allows for calculating displacement fields and the evolution of strain fields on the observed object’s surface. The translation, rotation, and shear transformations of each pixel’s positions before and after motion are calculated using expressions (2)–(4) [57].

(2) $$\varepsilon_{x}=\frac{\partial u}{\partial x}+\frac{1}{2}[{\left(\frac{\partial u}{\partial x}\right)}^{2}+{\left(\frac{\partial v}{\partial x}\right)}^{2}]$$

(3) $$\varepsilon_{y}=\frac{\partial v}{\partial y}+\frac{1}{2}[{\left(\frac{\partial u}{\partial y}\right)}^{2}+{\left(\frac{\partial v}{\partial y}\right)}^{2}]$$

(4) $$\gamma_{xy}=\frac{1}{2}(\frac{\partial u}{\partial y}+\frac{\partial v}{\partial x}+\frac{\partial u}{\partial x}\frac{\partial u}{\partial y}+\frac{\partial v}{\partial x}\frac{\partial v}{\partial y})$$

where *ɛ_x_*, *ɛ_y_*, and *γ_xy_* represent distinct strain components, and *u* and *v* denote displacements in the *x* and *y* directions, respectively.

The DIC depends on the high-contrast identifiable optical characteristics attached to the surface. As the attachment point deforms, the identifiable optical characteristics will move. Therefore, a thin and uniform white background was sprayed on the surface of CASS specimens after cleaning the impurities on the surface of CASS specimens. Then, the black non-repeating, random, and isotropic speckles were scattered on the front surface of CASS disc specimens, as shown in the specimen magnification diagram of Figure 4b. During the experiment loading, image acquisition was conducted at a constant rate of 1 frame per second (fps) with a resolution of 2048 × 2048 pixels.

## 3. Experiment Results and Discussion

### 3.1. The Optimal Mix Proportion of CASS

**Figure 5:**
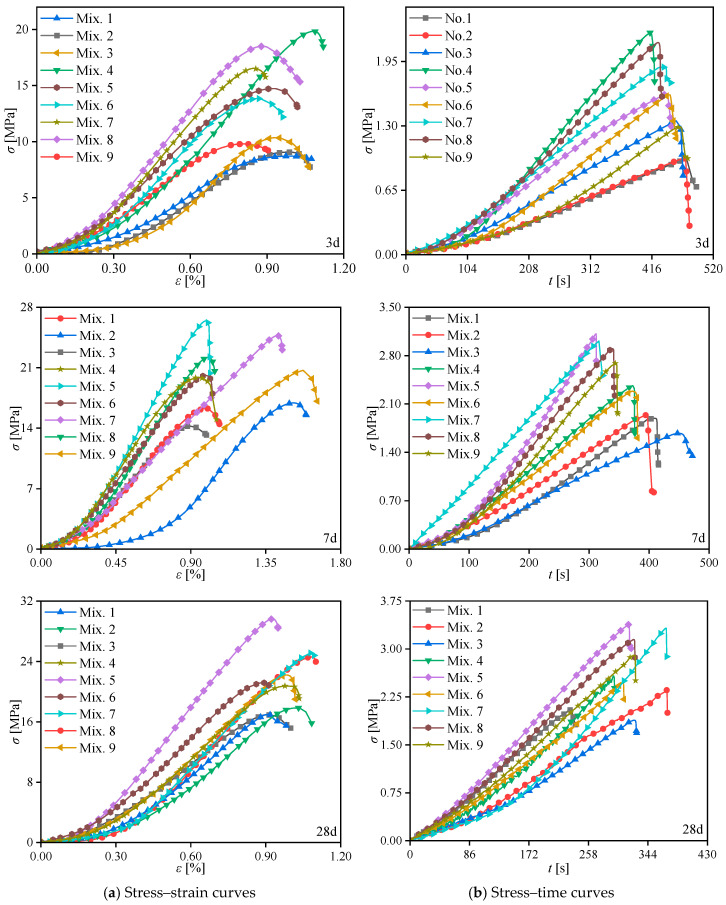
Stress–strain and stress–time curves of CASS with various mix proportions at 3 d, 7 d, and 28 d curing age under uniaxial compression and Brazilian splitting tests.

The stress–strain and stress–time curves for CASS, exhibiting various mix proportions at curing ages of 3 d, 7 d, and 28 d under uniaxial compression and Brazilian splitting tests, are illustrated in Figure 5. A visual inspection of the curves reveals a distinct trend in the mechanical behavior of CASS across different curing stages. Notably, the stress–strain curves exhibit a characteristic nonlinearity, indicative of the material’s ductile behavior at early stages, which transitions to a more brittle response as the curing age increases. This shift towards brittleness is evidenced by the steeper slope in the post-peak regime of the stress–strain curves for the 28-day curing age, suggesting a reduction in the material’s capacity for plastic deformation. The stress–time curves from the Brazilian splitting tests further substantiate this observation, with a more rapid decline in load-bearing capacity observed in the specimens with higher curing ages, highlighting an increased susceptibility to tensile failure. Observations of the peak compressive/tensile stress for CASS specimens with nine different mix proportions at 3 d, 7 d, and 28 d curing ages are presented in Table 7, based on Figure 5. It can be seen that strength values generally increase with the curing age, reflecting the improved bond strength and material cohesion over time. However, the increase in strength is more pronounced at earlier curing ages, with a subsequent tapering off at 28 days, indicating a maturation in the material’s strength.

To comprehensively explore the impact of four factors in orthogonal experiments—cementitious material content, water–binder ratio, sand ratio, and accelerator amount—on the compressive and tensile strength of CASS specimens, a range analysis using the data from Table 7 was conducted. Table 8 provides the results of the range analysis, where the R-value indicates the extent of variation in test indicators with changing influencing factors. Essentially, the R-value serves as an indicator for determining primary and secondary relationships among these factors. Notably, in Table 8, when assessing the impact of factors on compressive and tensile strength at curing ages of 3 d, 7 d, and 28 d, the influence of the accelerator amount on both strength parameters gradually diminishes with increasing curing age. For compressive strength, the cementitious material content and the water–binder ratio are the primary factors, with decreasing influence observed with the accelerator amount as the curing age increases. This trend underscores the importance of an appropriate balance between these components for achieving optimal strength. Similar trends are observed for tensile strength, with the sand ratio also emerging as a significant factor, particularly at 7 and 28 days of curing. These findings are instrumental in guiding the mix design process for CASS, emphasizing the need for a multifactorial approach to optimize the material’s static mechanical properties.

**Figure 6:**
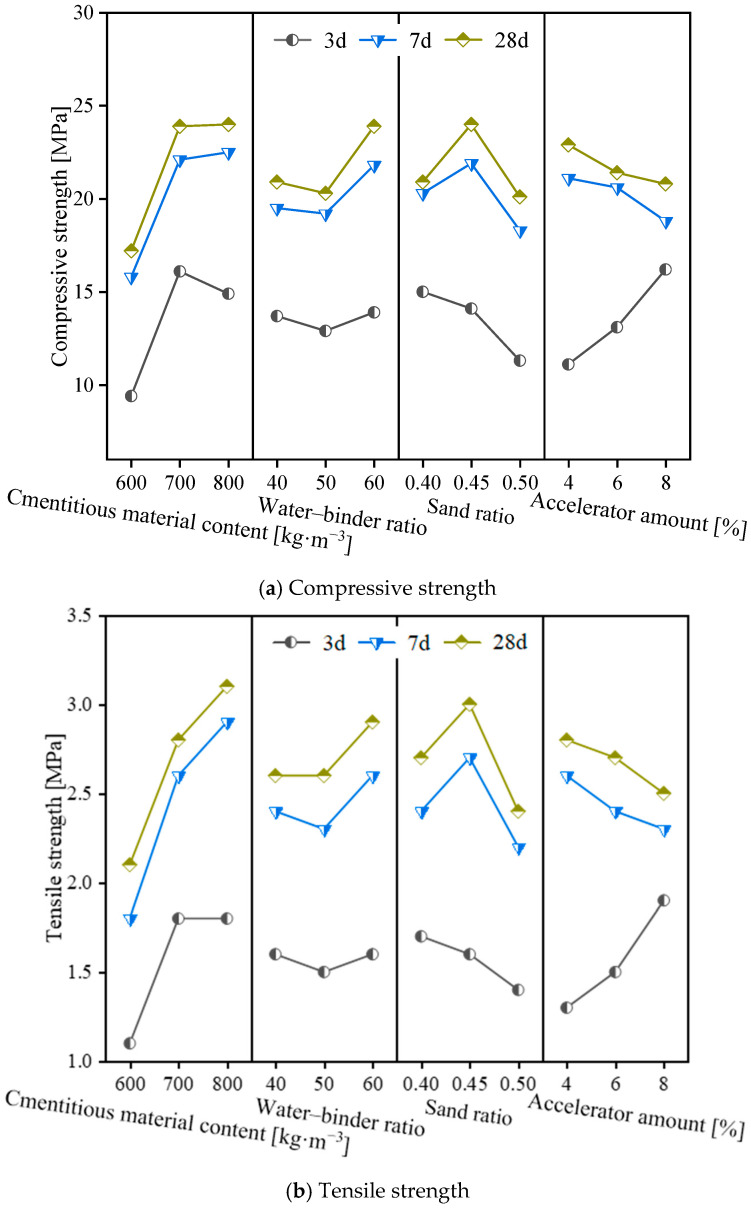
The relationship between the compressive/tensile strength of CASS and the variation in cementitious material content, water–binder ratio, sand ratio, and accelerator amount at different curing ages.

The variation in both compressive and tensile strength of the coral aggregate seawater shotcrete (CASS) specimens is illustrated in Figure 6, with respect to different curing ages and mix proportions. An increase in the content of cementitious material from 600 kg/m^3^ to 700 kg/m^3^ corresponded to a significant enhancement in compressive strength, amounting to 6.7 MPa, 6.3 MPa, and 11.7 MPa, and in tensile strength by 0.7 MPa, 0.8 MPa, and 0.7 MPa, for the curing ages of 3 d, 7 d, and 28 d, respectively. However, a further increase in the cementitious material content from 700 kg/m^3^ to 800 kg/m^3^ led to a decrease in compressive strength at the same curing ages by −1.2 MPa, 0.4 MPa, and 0.1 MPa, respectively, while the tensile strength exhibited an increment of 0 MPa, 0.3 MPa, and 0.3 MPa. These findings indicate that there exists an optimal threshold for the cementitious content, beyond which additional increments do not yield a proportionate enhancement in the concrete’s strength.

Figure 6 reveals that increasing the water–binder ratio from 0.4 to 0.45 decreased compressive and tensile strength at 3 d curing age by 6%, 24.7%, 5.9%, and 17.6%. For specimens cured for 7 d and 28 d, the compressive and tensile strength apex was attained at a water–binder ratio of 0.45, with a subsequent decline observed as the ratio was adjusted to 0.4 and 0.5. Specifically, at a 7 d curing stage, a reduction in compressive and tensile strength of 7.3%, 16.4%, 11.1%, and 18.5% was recorded. This trend continued at a 28 d curing stage, with 12.9%, 16.3%, 10%, and 20% noted decreases. These results indicate that, after reaching a certain curing age, strength initially increases with an increased water–binder ratio but eventually decreases.

For the change rule of strength with sand ratio, compressive and tensile strength remains relatively stable at lower sand ratios of 40% and 50%. However, a 60% sand ratio increase significantly enhanced compressive and tensile strengths by approximately 10% at both the 7 d and 28 d curing stages. This improvement is attributed to the higher sand ratio, which fosters a more homogeneous and densified concrete microstructure, an effect that is less pronounced at the lower sand ratios.

Contrary to the water–binder ratio, the accelerator amount enhances early strength. It shows a positive correlation between compressive/tensile strength at 3 d curing age and the accelerator amount. Specifically, 6% and 8% increase results in a respective 18%, 45.9%, 15.4%, and 30.8% augmentation in compressive and tensile strength. However, at 7 d and 28 d curing ages, there is a negative correlation, with compressive and tensile strength decreasing by 2.4%, 10.9%, 7.7%, and 11.5% at 7 d and 6.6%, 9.2%, 3.6%, and 10.7% at 28 d, respectively. This suggests that a high accelerator amount reduces compressive strength after a specific curing period.

In summary, the CASS mix proportions exert a similar influence on the tensile/compressive strength of specimens. Considering compressive and tensile strength across all curing ages, the most effective mix proportion comprises 700 kg/m^3^ of cementitious materials content, a water–binder ratio of 0.45, a 60% sand ratio, and an 8% dosage for the accelerator amount. Subsequently, an analysis of the static mechanical properties of specimens with this optimal mix proportion was conducted.

### 3.2. Strength and Failure Behavior under Uniaxial Compression

**Figure 7:**
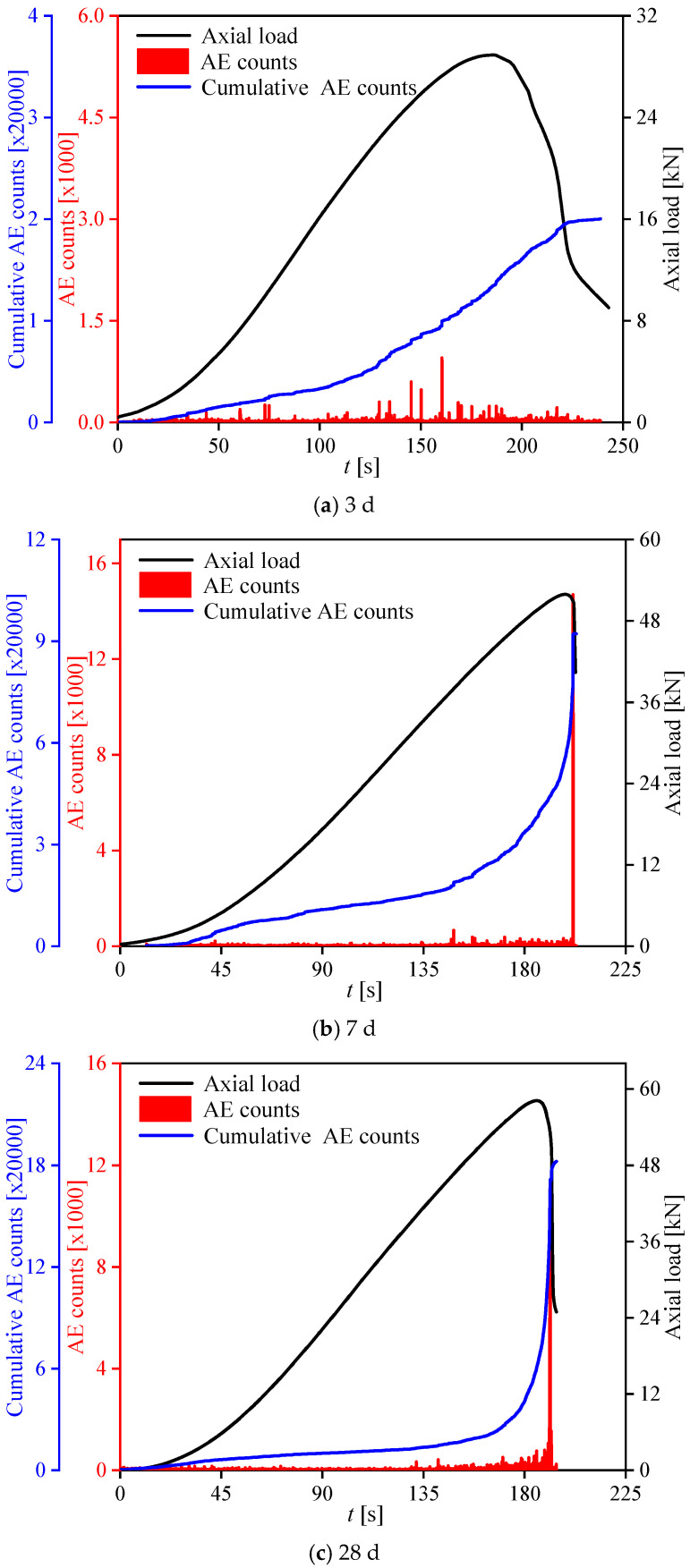
Axial load AE count curves of CASS at various curing ages.

When combining the concurrently measured outcomes from the DNS load transducer and AE sensor affixed to the specimens, Figure 7 portrays the nonlinear stress–strain relationships and axial load AE count curves for CASS with optimal mix proportions under uniaxial compression at various curing ages. As illustrated in Figure 7a–c, continuous weak AE signals signifying the initiation of microcracks were identified during pre-peak stages. The cumulative count gradually increased, while the instantaneous count remained relatively low in this period. However, as macrocracks began to form, a sudden surge in AE counts occurred around the axial load peak, leading to a rapid rise in cumulative counts within a short duration. Additionally, the more pronounced phenomenon with the advancement of curing age indicates the increasing brittleness of CASS specimens.

The complete compressive stress–strain curves of CASS at different curing ages can be categorized into four stages: consolidation, elastic, plastic, and fracture. The consolidation stage highlights the porous and loose characteristics of the coral concrete microstructure, with the curve exhibiting an initial concave rise due to compaction of the pore structure of coral aggregate and voids within the concrete matrix under increasing loading stress. Subsequent stages—elastic, plastic, and fracture—are akin to the characteristics of brittle materials like rock, attributed to the relatively low compressive strength and brittle nature of coral itself.

Independent of what kind of micro-fracture forms in brittle materials, it is accompanied by the release of strain energy stored in brittle materials and propagated in the form of elastic waves at the moment of its occurrence, which is AE. Different micro-fracture behaviors correspond to different AE information characteristics. Therefore, this study obtained the tensile–shear properties of brittle materials by judging the difference of AE information, discussed the evolution process and law of crack development induced by tensile stress and shear stress at the meso-level, and further explained the internal tensile–shear failure mechanism of materials.

**Figure 8:**
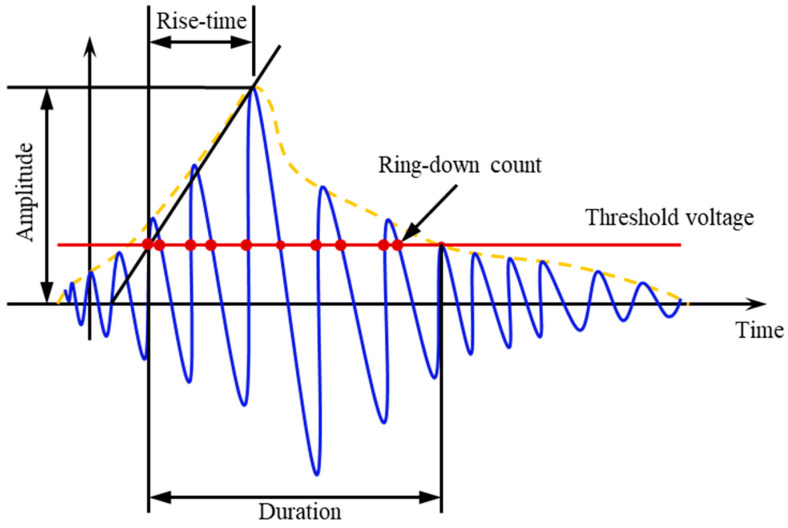
Schematic figure of the characteristic parameter definition of the acoustic emission signal.

Studies in this domain have revealed that micro-fracture AE signals’ RA value (rise time/amplitude) and AF value (ring count/duration time, average frequency) can serve as indicators of brittle material crack tensile or shear properties, facilitating further classification of micro-fracture forms [58]. The characteristic parameters of AE signals are visually represented in Figure 8. Generally, a low RA value and high average frequency in the AE signal suggest micro-fractures induced by tensile stress surpassing the cementation’s tensile strength, indicating a tensile crack. Conversely, a high RA value and low average frequency indicate micro-fractures caused by shear stress exceeding the cementation’s shear strength, signifying a shear crack. The determination of the relative RA value and AF value ratio is crucial for understanding the cause of microcrack initiation. Nevertheless, in current research, there is no clearly defined ratio between RA value and average frequency as a criterion for distinguishing tensile and shear cracks.

**Figure 9:**
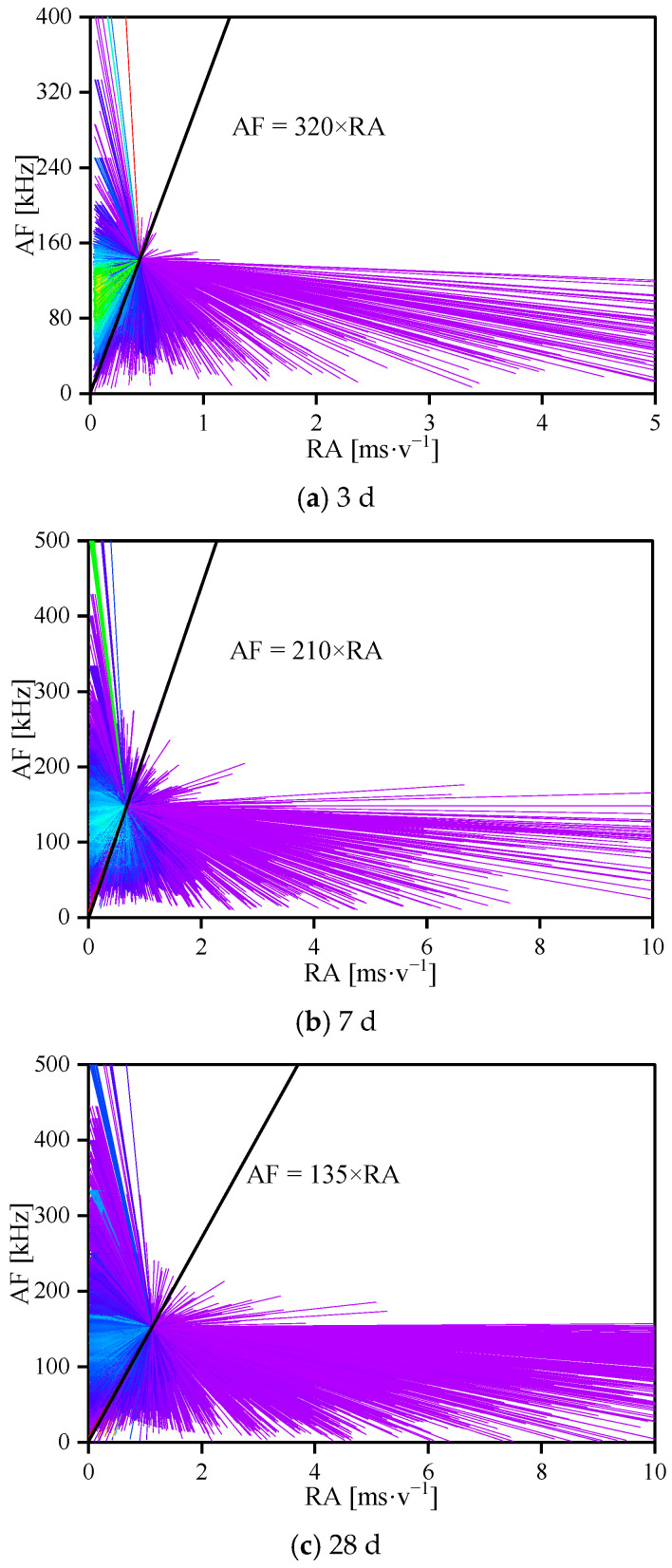
The cluster analysis results of acoustic emission RA-AF values of CASS at various curing ages.

Drawing on the concept that the ratio of RA value to AF value during tensile–shear crack initiation falls within a relatively stable interval, K-means clustering analysis, employing a minimization criterion, was utilized for statistical classification of AE data. This approach deepened exploration into the concealed micro-fracture formation mechanism in CASS specimens. Figure 9 illustrates the cluster analysis outcomes of acoustic emission AF-RA values for CASS specimens with 3 d, 7 d, and 28 d curing ages.

As displayed in Figure 9, the dividing boundary between the final iterative convergence particle and the coordinate origin in the K-means clustering analysis serves as the demarcation line distinguishing the tensile crack and shear crack clusters. Points above the oblique line in the AE data denote occurrences of tensile micro-fractures, while points below signify shear micro-fractures. The cluster analysis results reveal that AE signals with higher average frequency and lower RA value primarily manifest in the initial loading stage. Subsequently, as the load intensifies, lower AF value and RA value gradually increase, paralleled by a rise in shear cracks. Typically, shear crack formation ensues after the initiation, propagation, and evolution of tensile cracks, leading to significant damage to the internal structure of concrete specimens, compromising their integrity and eventual failure. In comparison to specimens aged 3 d, those aged 7 d and 28 d exhibit increased occurrences of tensile cracks in the early loading stages. As tensile cracks accumulate, shear cracks proliferate rapidly, culminating in the specimens’ instability and failure. This underscores that tensile failure constitutes the primary mechanism behind the ultimate instability of concrete specimens. As curing age increases, CASS specimens tend towards a more brittle material nature, and the severity of brittle and shear failure intensifies upon subjecting the specimens to higher loads.

### 3.3. Strength and Failure Behavior under Splitting Tensile

Determining tensile strength traditionally involves a direct uniaxial tensile test. However, the Brazilian splitting tensile test, an indirect method outlined in the ASTM standard [59], presents notable advantages in terms of practicality, simplicity, and cost-effectiveness. This test gains significance when engineers encounter intricate stress fields, combining compressive and tensile stresses in material mechanic design. Accurately representing field conditions necessitates considering tensile strength in the presence of compressive stresses. The splitting tensile strength test emerges as a straightforward method for simulating compression-induced stress fields.

Besides its practicality, the splitting tensile strength test is commonly employed to estimate compressive strength. Birid [60] established a correlation between compressive strength and splitting tensile strength test results. Incorporating parameter adjustments based on related research, the equation was adapted to predict CASS tensile strength by compressive strength. This modification demonstrated comparable accuracy in predicting tensile strength:

(5) $$\frac{UCS}{ST}=6.3\frac{\pi }{2}{\left(\frac{H}{D}\right)}^{0.2}$$

where *H* is the height, and *D* is the diameter of the specimen for the STS test.

**Figure 10:**
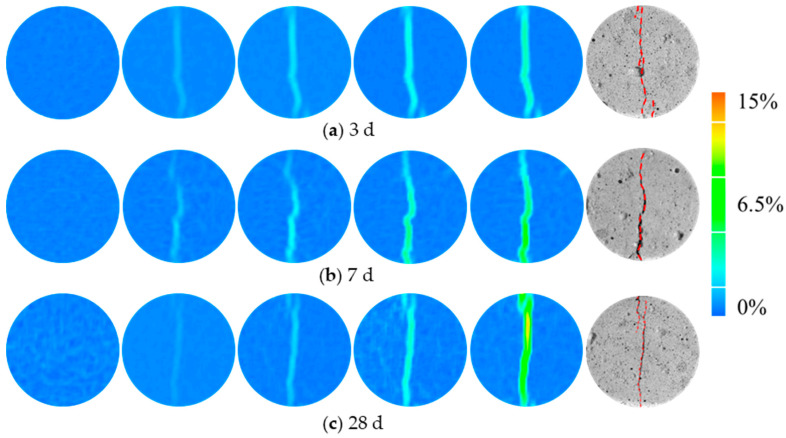
Failure pattern and major principal strain nephograms of CASS specimens at various curing ages under Brazilian splitting tests.

The evolution of the major principal strain nephograms of the specimens can be seen in Figure 10. At the beginning of loading, the pore structure of coral aggregate and the voids inside the concrete matrix were relatively uniformly distributed in the plane of the disc resulting in a relatively uniform strain distribution. When the load reached peak pressure, a strain concentration zone appeared along the loading diameter, with a strain of about 2.6%. As the loading continues, the strain concentration zone expands. Correspondingly, the cracks extend from both ends to the center until the specimen loses its load-bearing capacity. It is noteworthy that the major principal strain of specimen in 28 d curing age reached about 14%, which was higher than that of 5% in 3 d and 7% in 7 d. This is due to the increasingly obvious brittle properties of CASS with the increasing curing age. As a whole, the Brazilian splitting failure is caused by the main crack that initiates from the ends and propagates along the loading line, and the main crack is arc-shaped.

### 3.4. Comparative Analysis of Static Mechanical Properties

**Figure 11:**
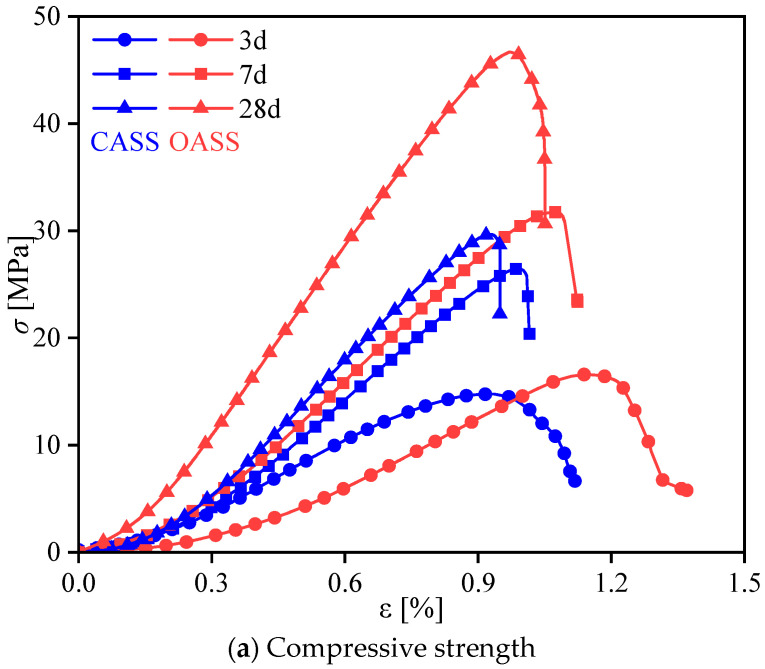

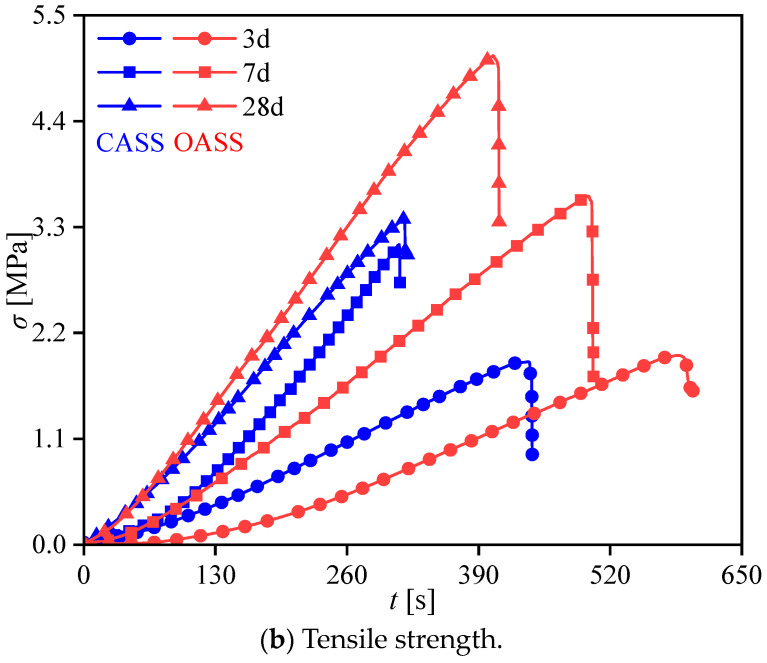
Comparison of strength at various curing ages between ordinary shotcrete and CASS.

To delve deeper into the static mechanical properties of CASS and verify the validity of mix proportion, this investigation conducted a comparative analysis between OASS and CASS. Both were subjected to identical specimen preparation methods, curing conditions, and mix proportions. Illustrated in Figure 11 is the nonlinear stress–strain relationship of OASS and CASS at various curing ages. Notably, CASS exhibited a swifter increase in early strength compared to conventional shotcrete, followed by a more gradual progression in later strength. Specifically, the strength of coral concrete surged by 40% and 44% from 3 d to 7 d concerning the 28 d strength. The subsequent development of the remaining 10% of ultimate curing strength spanned three weeks. Nevertheless, the later strength of CASS proved to be considerably lower than that of OASS.

**Figure 12:**
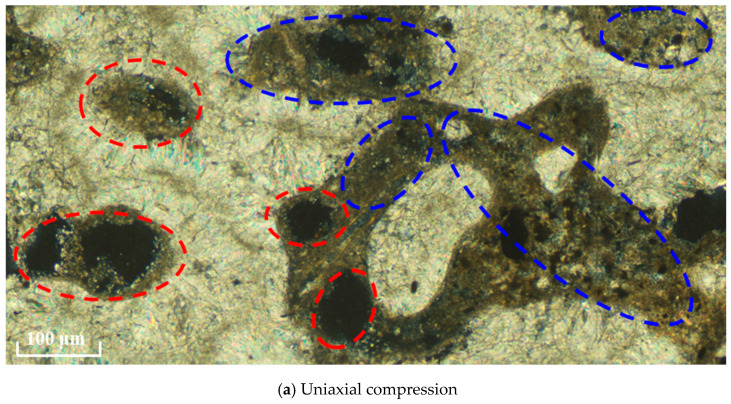

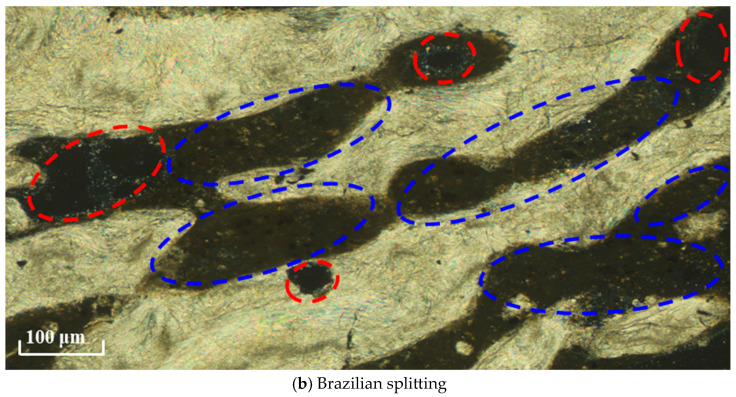
Microscopic enlarged figure of fracture surface after the failure of the laboratory test specimen, in which filling of small particle minerals and pore are identified by the blue dotted frame and red dotted frame, respectively.

On one side, the porous microstructure inherent in coral debris serves as a water reservoir, absorbing and retaining a specific quantity of water from the concrete slurry. This reservoir releases water during the hydration and hardening process. Conversely, the infusion of small-particle minerals in CASS into coral pores enhances the embedding of cementitious material and aggregate, as illustrated in the blue box in Figure 12. This dual mechanism not only heightens specimen compactness but also elevates early strength and elastic modulus. Nonetheless, the presence of unfilled pores (highlighted in the red box in Figure 12) emerges as water evaporates, causing dispersion of energy and deformation in specimens. This, in turn, diminishes the material’s strength and elastic modulus in the later stages. In summary, in comparison to OASS, the heightened early strength and elastic modulus in CASS result partly from the mineral-filling effect on coral pores. Simultaneously, the unfilled pores contribute to the reduced strength and elastic modulus in the later stages.

## 4. Numerical Simulation

The preceding study conducted quasi-static compression and splitting tests on CASS, briefly discussing the static mechanical properties coupled with DIC and AE techniques. Nevertheless, although laboratory tests present a dependable approach for investigating the macroscopic static mechanical behavior of CASS, the existing constraints of monitoring instrumentation impose significant challenges in comprehensively monitoring the micro-morphology and complete deformation damage process of the structural body at the micro-scale. As a result, a mesoscopic model in three dimensions based on PFC was developed for CASS, taking into account the arbitrary shapes, sizes, and distributions of aggregates. Verification of the model involved comparing the outcomes of quasi-static compression and splitting tests on cylindrical/disc specimens through both experimental and numerical analyses. The microcrack behavior of CASS was quantitatively analyzed at the micro-scale.

### 4.1. Modeling Process

**Figure 13:**
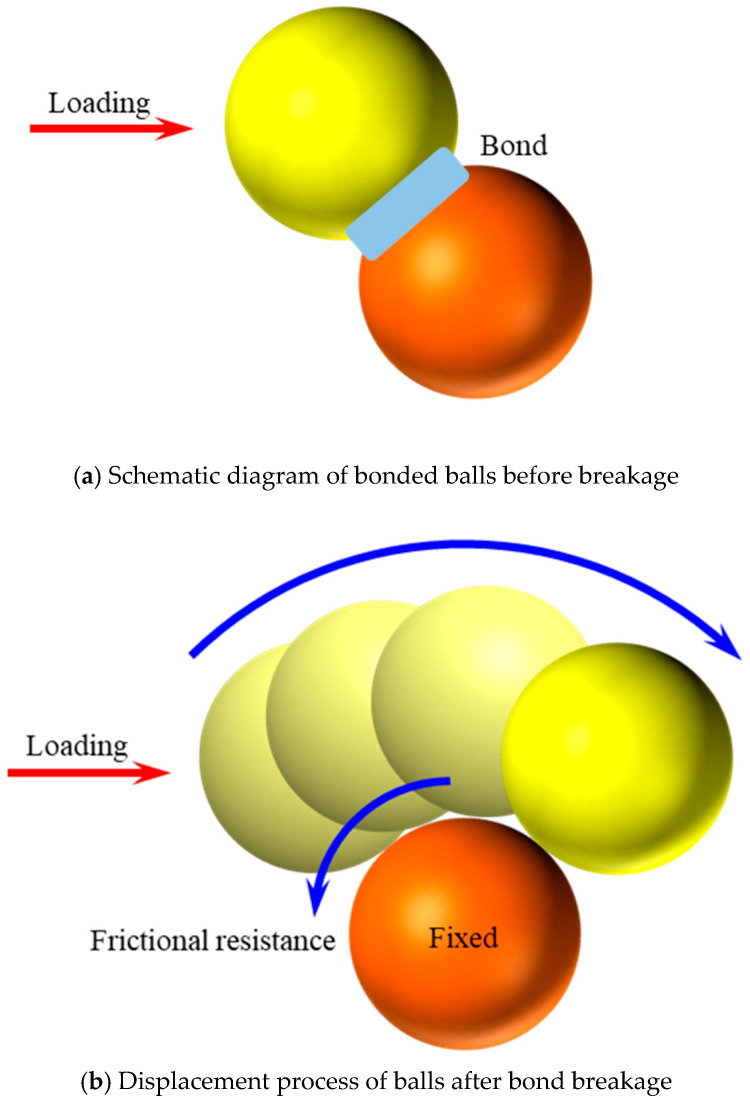
Structural and mechanical behavior of the PBM.

The parallel bond model (PBM) was employed as the contact model for particles in the simulation of CASS in this study as the first step of constructing the numerical model. The PBM implemented in this study incorporates linear elastic components and bonded parts at the contact interface, offering two distinct interfacial behaviors (refer to Figure 13a,b) [61,62]: (i) A minuscule, linearly elastic interface designed to transmit forces without tension and display frictional characteristics. This interface functions based on identical mechanical principles as the linear model, allowing relative rotation without resistance, with shear forces governed by a Coulomb limit. (ii) A finite-sized, linearly elastic, and bonded interface that can transfer both forces and moments. In cases where the interface is bonded, it resists the relative rotation of the particles on either side of the contact. However, the parallel bonding may fracture if the tensile strength and shear stress exceed the bonding material’s strength. The rupture of the parallel bond results in the loss of bonding material, accompanied by the dissipation of associated forces, moments, and stiffness.

Considering the porous characteristics of both concrete and coral aggregates and aiming to represent the enhanced compactness of specimens by incorporating small particle minerals, this study separated silica fume from the cement mortar phase system during the modeling process. Then, the CASS was treated as a four-phase material comprising cement mortar, silica fume, coral debris, and the interfacial transition zone. Within this study, a discrete element model was constructed to represent the mixed materials by generating two types of units: balls and clusters. The ball units were employed to characterize the shape of the smallest constituent unit of each material phase, while the cluster units were employed to characterize the coral debris component.

**Figure 14:**
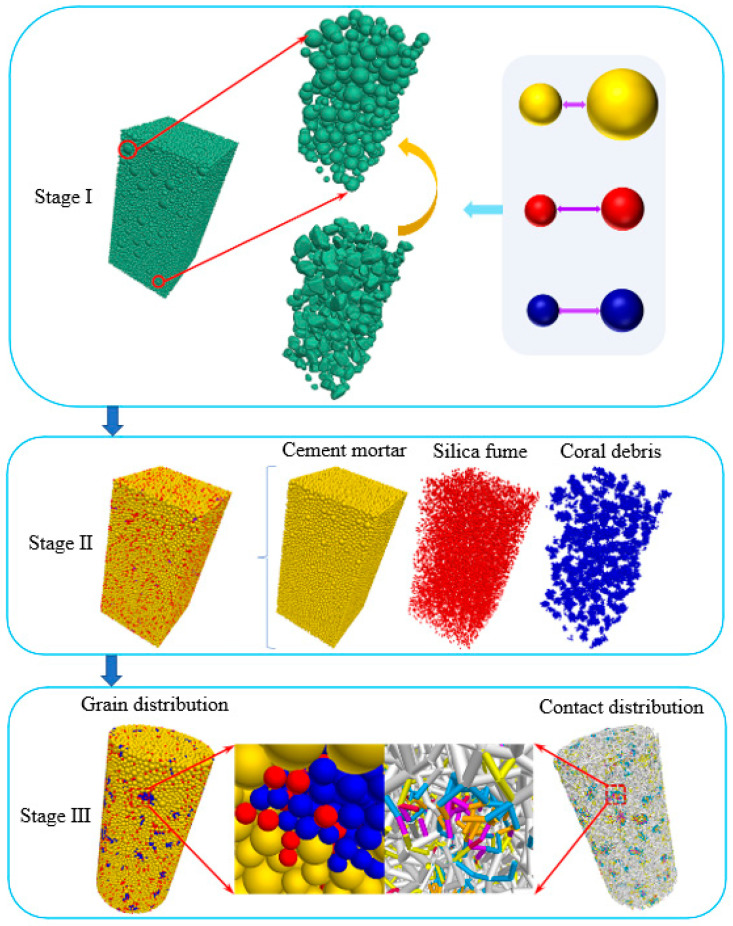
The figure of the modeling process.

Figure 14 shows the modeling process of the PFC as follows:

(1) Stage I: To reproduce laboratory findings, we selected, categorized, and 3D-scanned corals used for mixing in PFC. Initially, a concrete specimen model with only spherical particles was created based on the established gradation. Subsequently, larger particles were randomly replaced with coral debris shapes based on volume equivalence. This process culminated in a realistic concrete structure model, which was preloaded with isotropic stress to simulate initial conditions.
(2) Stage II: Mineral components were divided according to the concrete structure model, CASS mix proportions, and grading curves. Red particles, sized between 0.45 and 0.6 mm, symbolized silica fume, while yellow particles of 0.6 to 1 mm diameter represented cement mortar. The coral debris in the model was filled with blue particles of 0.45 to 0.6 mm. The model also incorporated the random distribution of red silica fume particles within coral clusters to enhance specimen compactness, as detailed in the enlarged view of Figure 14 (Stage III).
(3) Stage III: Models were constructed with dimensions matching laboratory specimens (Φ50 × 100 mm and Φ50 × 25 mm) by refining the Stage II model. Contact between different particles was accounted for, assigning different values. Figure 14 (Stage III) shows the contact distribution, color-coded to indicate six distinct contact types: cement mortar to cement mortar (F-F), silica fume to silica fume (S-S), coral debris to coral debris (C-C), cement mortar to coral debris (F-C), cement mortar to silica fume (F-S), and coral debris to silica fume (C-S). The micro-parameter assignment endowed the numerical specimens with defined bearing strength, finalizing the CASS model building.

### 4.2. Calibration of Micro-Parameters

**Figure 15:**
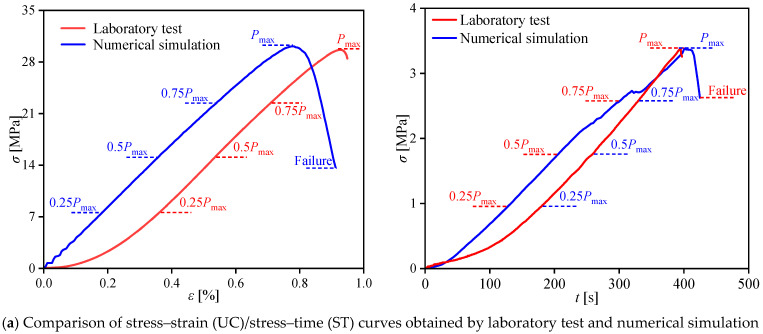

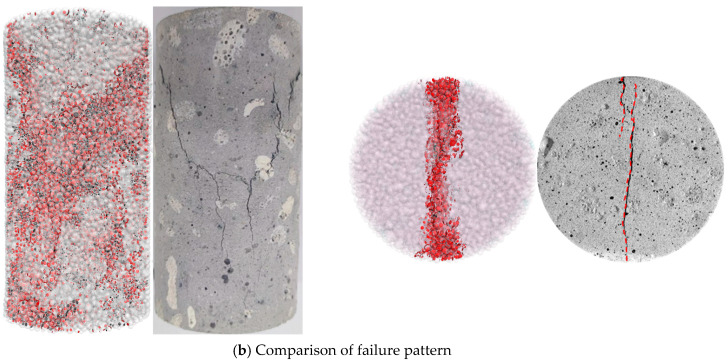
Comparison of numerical results based on PFC and experimental results in uniaxial compression (UC) and Brazilian splitting tests (ST).

Only the specimen with optimal mix proportion at 28 d curing age was subjected to numerical analysis in this investigation to mitigate the intricacy of the task and guarantee the practical implications of the numerical analysis computations. Currently, the “trial-and-error” method is prevalently implemented in the micro-parameter calibration of discrete element simulation specimens, and it has exhibited good outcomes [63,64,65]. The micro-parameters of the specimens calibrating results are shown in Table 9. A comparison of the specimens’ uniaxial compression and Brazilian splitting test results with the simulation findings presented in Figure 15 reveals that the stress–strain simulation curves highly agreed with the laboratory test curves. Particularly, the numerical simulation and laboratory test values of some key macroscopic mechanical parameters, including the specimens’ uniaxial compressive strength, splitting tensile strength, and elastic modulus, were approximately equal, indicating that the calibrated micro-parameters were reasonable.

### 4.3. Simulated Results and Discussion

In the model, two scale failure modes were simulated. First, a micro-scale crack was generated when a contact fracture occurred between two adjacent basic elements. According to fracture mechanics, the generated micro-scale cracks were divided into tensile cracks (TC) and shear cracks (SC). All symbolic representations of crack types and their percentage are listed in Table 10. With the increasing number of cracks, macro-scale fractures formed.

**Figure 16:**
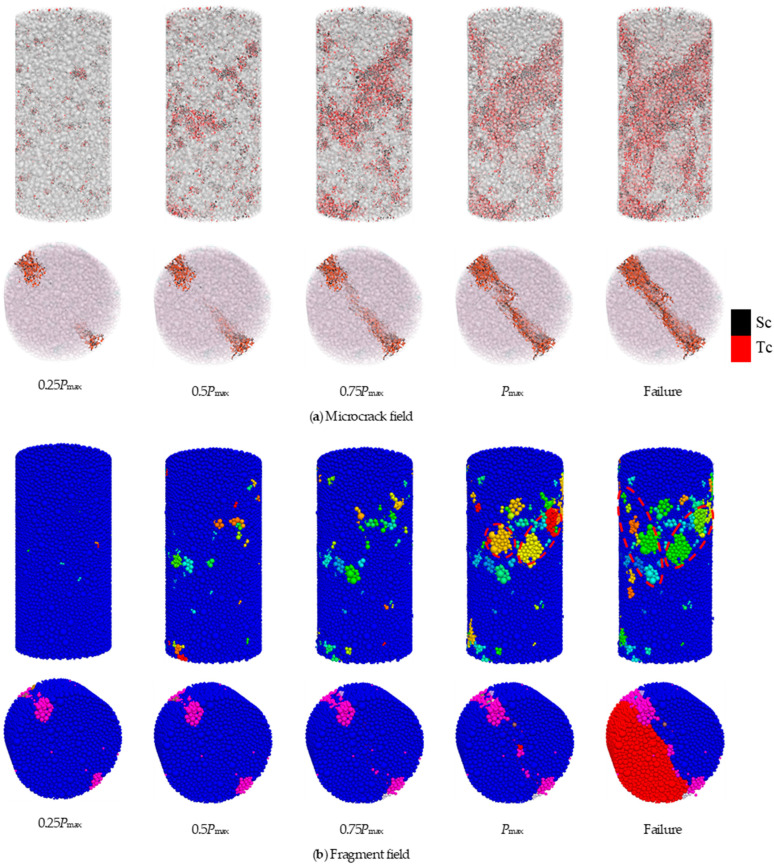
Numerical static damage accumulation process in uniaxial compression and Brazilian splitting tests.

**Figure 17:**
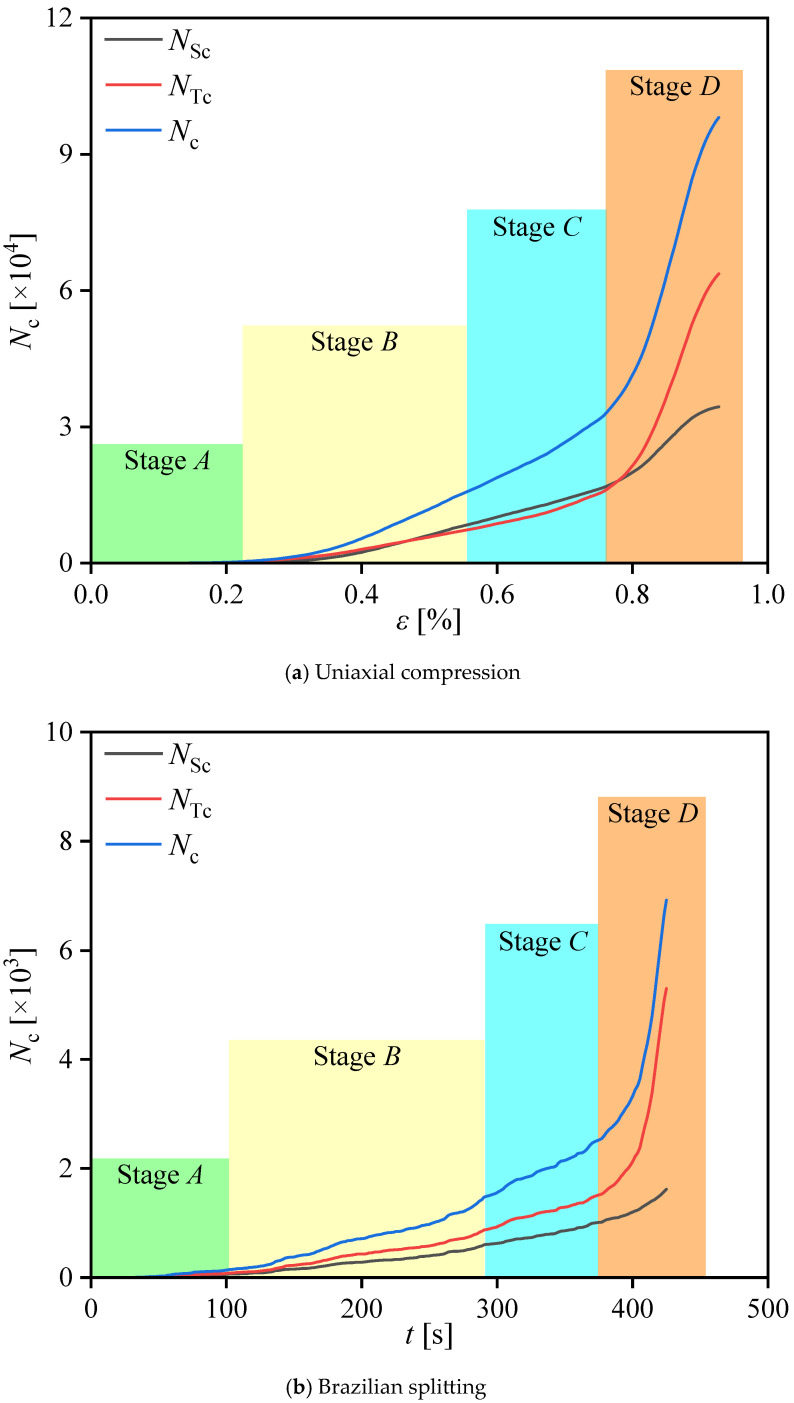
Variations in the number of cracks.

To facilitate subsequent analysis, we selected five representative moments throughout the loading process: 0.25*P*_max_, 0.5*P*_max_, 0.75*P*_max_, *P*_max_, and failure moment, where *P*_max_ is the peak load. According to the analysis of Figure 16 and Figure 17, the loading process of uniaxial compression and Brazilian splitting can be divided into the following stages:

Uniaxial compression:

(1) Stage A: With the load increasing from 0 to 0.25*P*_max_, exceeding the contact parameter, cracks and fragments emerge. During this stage, the integrity of the numerical specimen is comparatively superior;
(2) Stage B: In this stage, axial load increases from 0.25*P*_max_ to 0.5*P*_max_, and a small number of cracks and damage are observed within the numerical specimen, along with the sporadic appearance of fragments. However, the number of cracks and fragments retains a low level;

(3) Stage C: As the axial load increased from 0.5*P*_max_ to 0.75*P*_max_, the number of cracks inside the specimen further increased, the cracks in the numerical specimen began to hook up with each other and penetrate through, and the damage inside the model further accumulates;
(4) Stage D: With the axial load increased from 0.75*P*_max_ to *P*_max_, the number of cracks increased rapidly, and the large fragment field started to appear along the direction of the main crack extension, as shown by the red box in Figure 16b;
(5) Failure: The specimen transitioned into the macroscopic rupture phase, characterized by a steep increase in cracks and fragments. A large fragment field marked by red boxes occurred in the area of crack penetration;

Brazilian splitting:

(1) Stage A: With the load increasing from 0 to 0.25*P*_max_, the specimen began to be subjected to an external load. Stress concentration and fragment field began to form around the upper and lower loading points. Due to the low-stress concentration, only a small number of cracks and fragments occur;
(2) Stage B: The stress concentration around the loading points gradually increased with the load increasing from 0.25*P*_max_ to 0.5*P*_max_, resulting in the initiation of fresh cracks and the propagation of original cracks. Then, with the slow growth of the number of cracks and fragments, the high-stress concentration region inside the specimen gradually propagated from the loading points to the center;
(3) Stage C: As the load increased from 0.5*P*_max_ to 0.75*P*_max_, the crack propagation path and fragment field began propagating to the center of the specimens, forming the main crack path and fragment field.
(4) Stage D: When the load increases from 0.75*P*_max_ to *P*_max_, the load on the specimens reaches the maximum value. The relatively large number of cracks leads to significant particle displacement around the loading points, and the main crack gradually propagates to the specimen’s center. The specimen was on the threshold entry to the splitting failure stage, and the number of cracks and fragments increased relatively rapidly.
(5) Failure: Before the specimen was destroyed, the crack propagation rate and the number of cracks increased rapidly. At the moment of failure, the specimen was divided into two large fragments by the main crack propagating along the connection line between the upper and lower loading points.

**Figure 18:**
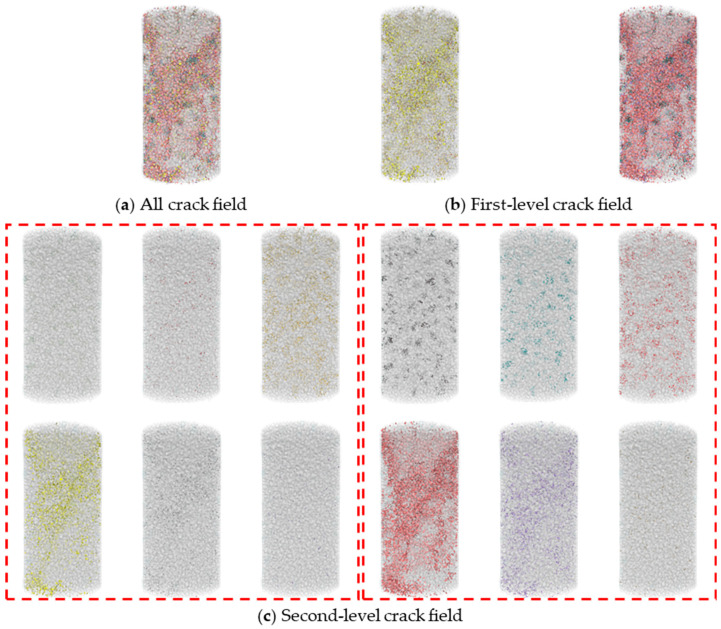
Multi-level crack division.

A multi-level characterization of cracks was developed in a static mechanical simulation based on PFC. Quantitative analysis was conducted on the cracks observed in the broken numerical specimen. Figure 18 provided an example of uniaxial compression where crack formations within a numerical model were divided into two levels. Figure 18a shows all cracks within the numerical specimen. Figure 18b further divided the cracks into tensile and shear, labeled as first-level cracks. Then, Figure 18c presented a further division of cracks into tensile/shear between various minerals and tensile/shear occurring within the same minerals, labeled as second-level cracks.

**Figure 19:**
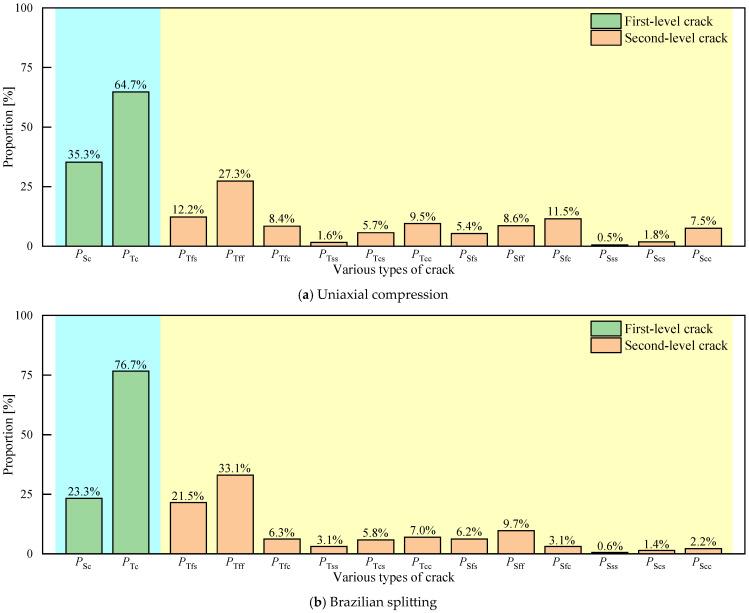
Proportions of the numbers of various cracks to the total cracks in uniaxial compression and Brazilian splitting after sample failure.

The quantities of various cracks presented in both uniaxial compression and Brazilian splitting were counted, as detailed in Figure 19. During the first-level cracks, the proportions of tensile/shear cracks to the total number of cracks in uniaxial compression and Brazilian splitting were 64.7%/35.3% and 76.7%/23.3%, which suggests that tensile failure was the main failure mechanism in uniaxial compression and Brazilian splitting. In second-level cracks of cylindrical specimens, the sum of *P*_Tfs_, *P*_Tff_, *P*_Tfc_, *P*_Tcs_, *P*_Sfs_, *P*_Sff_, *P*_Sfc_, and *P*_Scs_ is 80.9%. Identically, the sum in the disc specimen is 87.1%. That is because the CASS primarily experiences failure within the cement mortar and bonding surface between the cement mortar and aggregate (coral debris). Of particular note, in cylindrical specimens, the respective values of *P*_Tcc_ and *P*_Scc_ are 9.5% and 7.5%. The values of *P*_Tcc_ and *P*_Scc_ in the disc are 7% and 2.2%, which are not low. This can be attributed to the relatively weak physical and mechanical properties of coral debris in relation to other aggregates, which leads to a certain number of tensile and shear fractures of the coral debris upon the failure of concrete specimens. However, silica fume distributed between large-sized particles is a filling material with a relatively small particle size to improve the compactness of the specimen, which leads to little contact within silica fume and is also the reason why the number of tensile and shear cracks within silica fume is the least.

**Figure 20:**
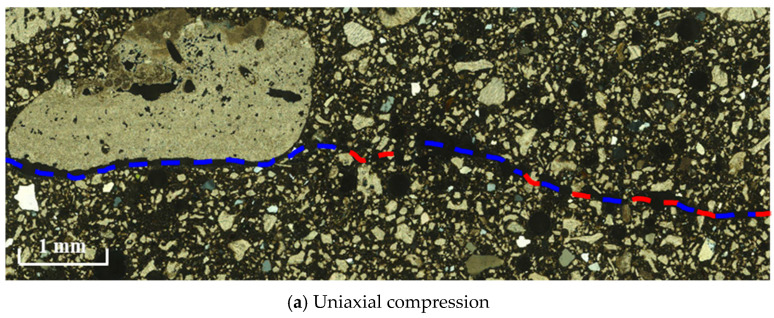

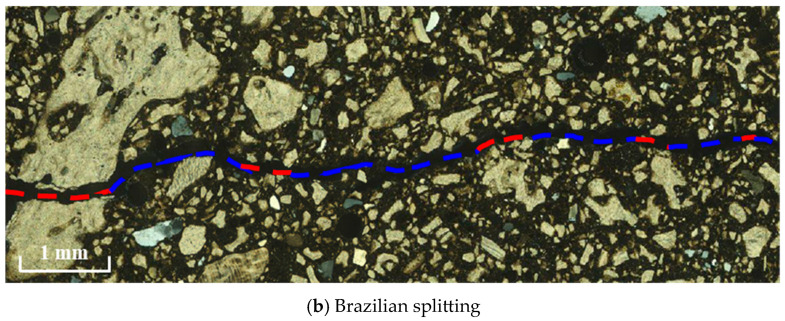
Experimental observation results of the microstructure of the real CASS specimen after failure, in which cracks within the cement mortar and bonding surface between the cement mortar and aggregate and cracks within other mineral components are identified by the blue dotted line and red dotted line, respectively.

Figure 20 shows the optical microscopic observations of the microstructure in a real CASS specimen after loading. As can be seen, along the macro fracture path, the number of cracks within the cement mortar and bonding surface between the cement mortar and aggregate (identified by a blue dotted line) is much larger than that of cracks within other mineral components (identified by red dotted line), which is consistent with the numerical results. This agreement verifies the reliability of the model.

## 5. Conclusions

This study investigated the static mechanical properties of CASS. The orthogonal experiments, which consisted of four-factor and three-level, were employed to explore the optimal mix proportion of CASS. On a macro-scale, uniaxial compression with the AE technique and splitting tests with the DIC technique were conducted on CASS at different curing ages using an electro-hydraulic servo-controlled test machine. The OASS was introduced to validate the effectiveness of the mix proportions for CASS by conducting macro-mechanical tests on two types of samples with identical mix proportions but differing in aggregate types. Meanwhile, on a micro-scale, the microcracks of numerical specimens were multi-level divided, and the behavior of microcracks was quantitatively analyzed. The following main conclusions can be drawn:

(1) The optimal mix proportion of CASS consists of 700 kg/m^3^ of cementitious materials content, a water–binder ratio of 0.45, a sand ratio of 60%, and a dosage of 8% for the accelerator amount.
(2) Tensile failure is the primary failure mechanism of uniaxial compression and Brazilian splitting. Among them, the main failure form of the Brazilian split is the two ends crack initiation and spread to the central. Meanwhile, the specimens of CASS exhibit an increased brittleness with longer curing age.
(3) Compared to OASS, the CASS with an optimal mix proportion has approximately equal early strength but low later strength. The early strength of CASS increases rapidly, but the later strength increases slowly. However, the strength of OASS increases evenly. The filling and water bag effect on coral pores makes a high early strength of specimens, but the unfilled pores in the specimens are also the reason for the low later strength.
(4) A novel numerical three-dimensional model based on PFC was proposed to simulate the static mechanical properties of CASS. The multi-level division and quantitative description of cracks are realized. The failure in CASS under uniaxial compression and Brazilian splitting tests is primarily experienced within the cement mortar and bonding surface between the cement mortar and aggregate (coral debris).

In conclusion, the study of coral aggregate seawater shotcrete (CASS) has revealed promising properties for its application in construction. The material’s early strength development and its environmental benefits, stemming from the use of coral as an aggregate, make it a sustainable option for construction in coastal and marine environments. However, consideration must be given to its performance at later stages of curing, which, while showing a slower increase in strength, still meets the requirements for many construction applications. Combined with understanding its static mechanical properties and failure mechanisms, the optimal mix proportion determined in this study provides a foundation for the potential use of CASS in structural support and other construction elements. Future research should focus on long-term performance and durability testing to fully assess its viability in construction practices.

## Figures and Tables

**Table 1:** Physical properties of coral debris.

Bulk Density (kg/m^3^)	Apparent Density (kg/m^3^)	Water Absorption (%)	Porosity (%)	Cylindrical Compressive Strength (MPa)
894	1973	12.52	48.97	2.69

**Table 2:** Physical properties of coral sand.

Fineness Modulus	Bulk Density (kg/m^3^)	Apparent Density (kg/m^3^)	The Saturated Surface Dry Moisture Content (%)
2.88	1138	2751	1.96

**Table 3:** Chemical composition of artificial seawater.

NaCl (kg/m^3^)	MgCl·6H_2_O (kg/m^3^)	Na_2_SO_4_ (kg/m^3^)	CaCl_2_ (kg/m^3^)	KCl (kg/m^3^)
24.5	11.1	4.1	1.2	0.7

**Table 4:** Orthogonal test table.

Mix	Cementitious Material Content (kg/m^3^)	Water–Binder Ratio	Sand Ratio (%)	Accelerator Amount (%)
Mix. 1	600	0.4	40	4
Mix. 2	600	0.45	50	6
Mix. 3	600	0.5	60	8
Mix. 4	700	0.4	50	8
Mix. 5	700	0.45	60	4
Mix. 6	700	0.5	40	6
Mix. 7	800	0.4	60	6
Mix. 8	800	0.45	40	8
Mix. 9	800	0.5	50	4

**Table 5:** Mix proportion of CASS.

Mix	Cementitious Material Content (kg/m^3^)	Cement (kg/m^3^)	Seawater (kg/m^3^)	Coral Sand (kg/m^3^)	Coral Debris (kg/m^3^)	Water Reducer (kg/m^3^)	Accelerating Agent (kg/m^3^)
Mix. 1	600	552	240	541	812	12	24
Mix. 2	600	552	270	655	655	13.5	36
Mix. 3	600	552	300	760	506	15	48
Mix. 4	700	644	280	590	590	14	56
Mix. 5	700	644	315	702	468	15.7	28
Mix. 6	700	644	350	448	672	17.5	42
Mix. 7	800	736	320	627	418	16	48
Mix. 8	800	736	360	395	395	18	64
Mix. 9	800	736	400	489	489	20	32

**Table 6:** The slump and rebound rate of CASS.

Parameter	Mix. 1	Mix. 2	Mix. 3	Mix. 4	Mix. 5	Mix. 6	Mix. 7	Mix. 8	Mix. 9
Slump (mm)	90	105	125	120	135	165	125	155	190
Rebound rate (%)	16.99	11.7	12.92	11.72	7.04	8.22	14.61	9.67	14.97

**Table 7:** Peak compressive stress (CS) and tensile stress (TS) table.

	Mix	Curing Age		
		3 d	7 d	28 d
CS (MPa)	Mix. 1	8.70	16.28	16.97
	Mix. 2	9.06	16.92	17.83
	Mix. 3	10.35	14.23	16.91
	Mix. 4	19.84	19.86	20.75
	Mix. 5	14.73	26.44	29.63
	Mix. 6	13.84	20.10	21.17
	Mix. 7	16.53	24.72	25.11
	Mix. 8	18.50	22.21	24.59
	Mix. 9	9.79	20.69	22.19
TS (MPa)	Mix. 1	0.96	1.89	2.05
	Mix. 2	0.97	1.94	2.36
	Mix. 3	1.35	1.67	1.88
	Mix. 4	2.24	2.36	2.58
	Mix. 5	1.63	3.12	3.38
	Mix. 6	1.61	2.29	2.49
	Mix. 7	1.90	3.01	3.32
	Mix. 8	2.14	2.91	3.15
	Mix. 9	1.29	2.69	2.90

**Table 8:** Range table of influence factors of compressive strength (CS) and tensile strength (TS).

	Curing Age		Cementitious Material Content	Water–Binder Ratio	Sand Ratio	Accelerator Amount
CS	3 d	*K*_1_	9.4	15.0	13.7	11.1
		*K*_2_	16.1	14.1	12.9	13.1
		*K*_3_	14.9	11.3	13.9	16.2
		*R*	6.77	3.70	0.97	5.16
	7 d	*K*_1_	15.8	20.3	19.5	21.1
		*K*_2_	22.1	21.9	19.2	20.6
		*K*_3_	22.5	18.3	21.8	18.8
		*R*	6.73	3.52	2.64	2.37
	28 d	*K*_1_	17.2	20.9	20.9	22.9
		*K*_2_	23.9	24.0	20.3	21.4
		*K*_3_	24.0	20.1	23.9	20.8
		*R*	6.73	3.93	3.63	2.18
TS	3 d	*K*_1_	1.1	1.7	1.6	1.3
		*K*_2_	1.8	1.6	1.5	1.5
		*K*_3_	1.8	1.4	1.6	1.9
		*R*	0.73	0.28	0.12	0.62
	7 d	*K*_1_	1.8	2.4	2.4	2.6
		*K*_2_	2.6	2.7	2.3	2.4
		*K*_3_	2.9	2.2	2.6	2.3
		*R*	1.03	0.44	0.27	0.25
	28 d	*K*_1_	2.1	2.7	2.6	2.8
		*K*_2_	2.8	3.0	2.6	2.7
		*K*_3_	3.1	2.4	2.9	2.5
		*R*	1.03	0.54	0.30	0.24

*Note*: *K*_j_ denotes the average value of compressive strength at the level of each influence factor j; *R* denotes the K-value range for each influencing factor.

**Table 9:** Micro-parameters of numerical model.

Microparameters	F-F	S-S	C-C	F-S	F-C	C-S
Tensile strength (MPa)	32.76	32.76	18.72	32.76	18.72	18.72
Cohesion strength (MPa)	48	48	28.8	48	28.8	28.8
Bonding activation gap (mm)	0.01	0.01	0.01	0.01	0.01	0.01
Elastic modulus (GPa)	8.5	8.5	21.25	8.5	14.45	14.45
Stiffness ratio	1.3	1.3	1.3	1.3	1.3	1.3
Friction angle (°)	13	13	13	13	13	13

**Table 10:** All symbolic representation of crack types and their percentage.

	Crack Types	Symbol	Proportion Symbol
Tensile crack (Tc)	Cement mortar–Silica fume	Tfs	*P*_Tfs_
	Cement mortar–Cement mortar	Tff	*P*_Tff_
	Cement mortar–Coral debris	Tfc	*P*_Tfc_
	Silica fume–Silica fume	Tss	*P*_Tss_
	Coral debris–Silica fume	Tcs	*P*_Tcs_
	Coral debris–Coral debris	Tcc	*P*_Tcc_
Shear crack (Sc)	Cement mortar–Silica fume	Sfs	*P*_Sfs_
	Cement mortar–Cement mortar	Sff	*P*_Sff_
	Cement mortar–Coral debris	Sfc	*P*_Sfc_
	Silica fume–Silica fume	Sss	*P*_Sss_
	Coral debris–Silica fume	Scs	*P*_Scs_
	Coral debris–Coral debris	Scc	*P*_Scc_

## Data Availability

Data will be made available on request.
